# Role of Interleukins in Type 1 and Type 2 Diabetes

**DOI:** 10.3390/diagnostics15151906

**Published:** 2025-07-30

**Authors:** Roha Asif, Ammara Khalid, Tolga Mercantepe, Aleksandra Klisic, Sana Rafaqat, Saira Rafaqat, Filiz Mercantepe

**Affiliations:** 1 Department of Biotechnology, Lahore College for Women University, Lahore 44444, Pakistan; roha.asif@icloud.com (R.A.); amm_khan11998@yahoo.com (A.K.); sana.rafaqat44@gmail.com (S.R.); 2 Department of Histology, Faculty of Medicine, Recep Tayyip Erdogan University, Rize 53100, Türkiye; 3 Faculty of Medicine, Podgorica, Montenegro, University of Montenegro, 81000 Podgorica, Montenegro; aleksandranklisic@gmail.com; 4 Center for Laboratory Diagnostics, Primary Health Care Center, 81000 Podgorica, Montenegro; 5 Department of Zoology (Molecular Physiology), Lahore College for Women University, Lahore 44444, Pakistan; saera.rafaqat@gmail.com; 6 Department of Endocrinology and Metabolism, Faculty of Medicine, Recep Tayyip Erdogan University, Rize 53100, Türkiye; filiz.mercantepe@saglik.gov.tr

**Keywords:** interleukins, diabetes mellitus, inflammation, cytokines, type 1 diabetes, type 2 diabetes, immunopathogenesis, beta-cell dysfunction

## Abstract

**Background**: Despite distinct etiologies, type 1 diabetes (T1D) and type 2 diabetes (T2D) share chronic inflammation as a core feature. Interleukins, key immune mediators, play important yet still not fully understood roles in the development and complications of both conditions. **Objective**: This narrative review aims to provide a comprehensive and critical synthesis of current evidence on the role of key interleukins in T1D and T2D, highlighting their immunological functions, genetic associations, clinical correlations, and translational potential. **Methods**: A targeted literature search was conducted in PubMed, Google Scholar, and ScienceDirect up to January 2025, focusing on English-language clinical and experimental studies involving interleukins and their relevance to T1D and T2D. Reference lists were manually screened for additional sources. Interleukins (ILs) were reviewed individually to assess their immunobiology, disease specificity, and biomarker or therapeutic value. **Findings**: Pro-inflammatory cytokines such as IL-1β, IL-6, and IL-17 contribute to islet inflammation, insulin resistance, and microvascular damage in both T1D and T2D. Anti-inflammatory mediators including IL-4, IL-10, and IL-13 exhibit protective effects but vary in expression across disease stages. Less-characterized interleukins such as IL-3, IL-5, IL-9, and IL-27 demonstrate dual or context-dependent roles, particularly in shaping immune tolerance and tissue-specific complications such as nephropathy and neuropathy. Polymorphisms in IL-10 and IL-6 genes further suggest genetic contributions to interleukin dysregulation and metabolic dysfunction. Despite promising insights, translational gaps persist due to overreliance on preclinical models and limited longitudinal clinical data. **Conclusions**: Interleukins represent a mechanistic bridge linking immune dysregulation to metabolic derangements in both T1D and T2D. While their diagnostic and therapeutic potential is increasingly recognized, future research must address current limitations through isoform-specific targeting, context-aware interventions, and validation in large-scale, human cohorts. A unified interleukin-based framework may ultimately advance personalized strategies for diabetes prevention and treatment.

## 1. Introduction

Diabetes mellitus (DM) is defined as a group of metabolic disorders characterized by chronic hyperglycemia resulting from defects in insulin secretion, insulin action, or both. Diabetes is classified into type 1 diabetes (T1D) and type 2 diabetes (T2D). The World Health Organization (WHO) provides alarming statistics on diabetes prevalence, indicating a dramatic increase in cases globally, particularly in low- and middle-income countries. According to the International Diabetes Federation (IDF), around 537 million adults were living with diabetes in 2021, and this number is projected to rise significantly, underscoring the urgency of addressing diabetes as a public health crisis [1,2].

The pathophysiology of diabetes is multifaceted. In T1D, autoimmune destruction of pancreatic beta cells occurs, primarily driven by T lymphocytes [3]. In T2D, insulin resistance develops, often accompanied by beta-cell dysfunction, leading to chronic hyperglycemia. Inflammatory pathways are increasingly recognized as contributors to these processes. For example, studies have shown that chronic inflammation and elevated pro-inflammatory cytokines contribute to insulin resistance and beta-cell dysfunction in T2D [4].

**Figure 1:**
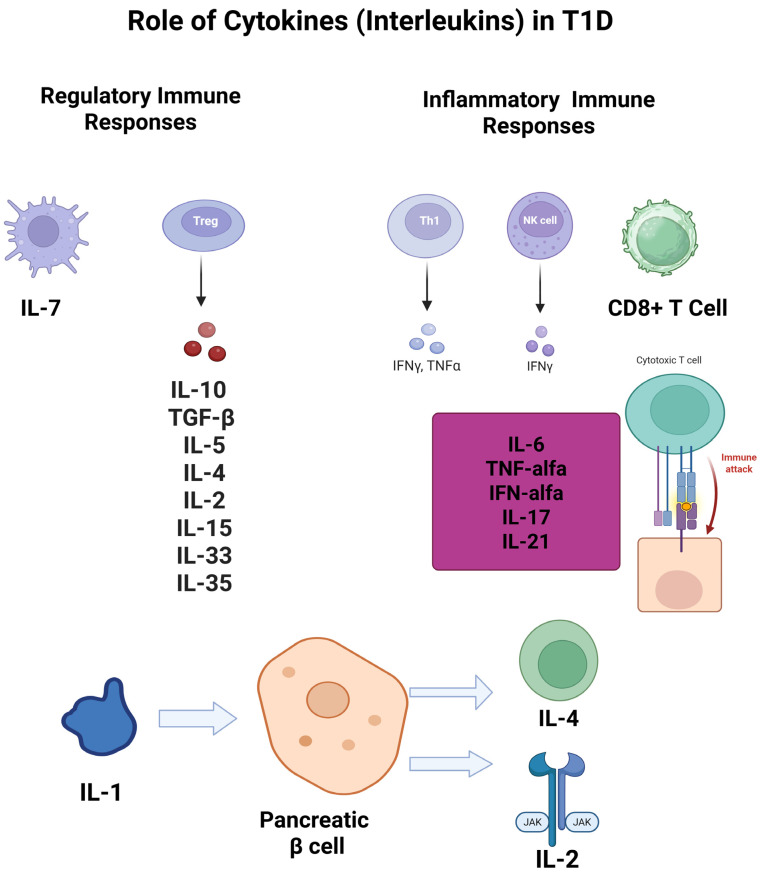
Role of Cytokines in Type 1 Diabetes (T1D): Cytokines secreted by immune and pancreatic cells have dual roles in the pathogenesis of T1D. On the left, regulatory cytokines (IL-10, TGF-β, IL-5, IL-4, IL-2, IL-15, IL-33, IL-35) and IL-7 produced by regulatory dendritic cells support anti-inflammatory immune responses and sustain regulatory T cells (Tregs). On the right, inflammatory cytokines (IL-6, TNF-α, IFN-α, IL-17, IL-21) promote the activation of pathogenic immune cells including Th1, Th17, CD8^+^ T cells, and NK cells. Pancreatic β cells, expressing receptors for several cytokines (IL-1, IL-4, IL-22), are particularly vulnerable to cytokine-mediated injury or regeneration. The interplay between these opposing signals influences both the initiation and progression of T1D.

The immune system plays a pivotal role in both types of diabetes, especially in the autoimmune nature of T1D. In T2D, low-grade inflammation, driven by immune mediators, is linked to insulin resistance [5]. Cytokines, particularly interleukins, have been shown to influence disease progression (explained in Figure 1) [6]. For instance, pro-inflammatory cytokines like interleukin-6 (IL-6) and tumor necrosis factor-alpha (TNF-α) are elevated in obesity and have been associated with insulin resistance and beta-cell dysfunction [7].

Interleukins are a subset of cytokines that play crucial roles in mediating immune and inflammatory responses. They facilitate communication between immune cells and are involved in various physiological processes, including the regulation of immune responses and inflammation. There are numerous interleukins, each with distinct roles in health and disease [8]. For instance, interleukin-1 (IL-1) is a key mediator of inflammatory responses, while interleukin-10 (IL-10) is known for its anti-inflammatory properties [9].

Recent research highlights the critical involvement of specific interleukins in diabetes pathogenesis. In T1D, IL-1β has been implicated in the destruction of pancreatic beta cells, and therapies targeting IL-1β have shown promise in preserving beta-cell function [10]. In T2D, interleukins like IL-6 and IL-18 contribute to insulin resistance and the inflammatory environment associated with obesity [11,12]. This growing body of the literature underscores the potential for targeting interleukins as therapeutic strategies in managing diabetes.

**Figure 2:**
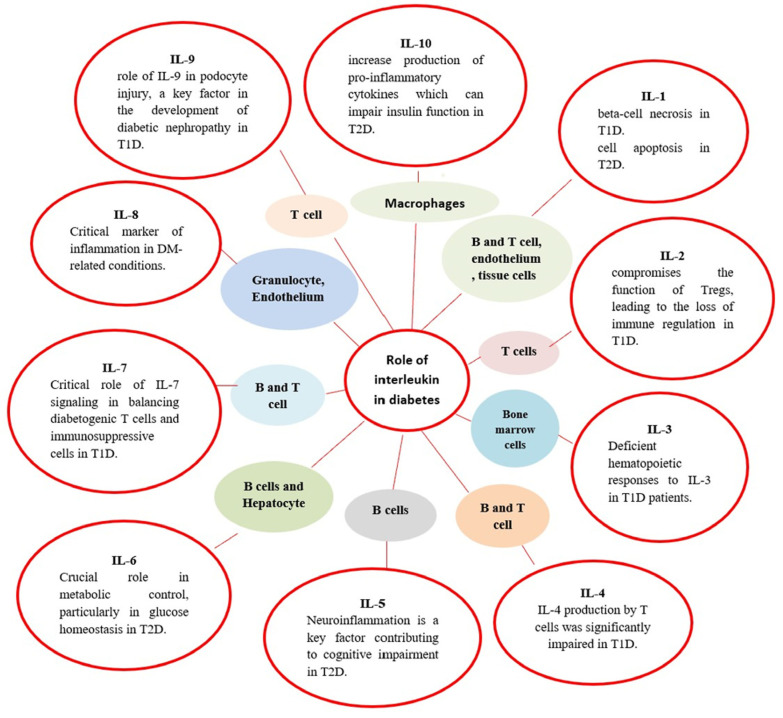
Pathogenesis of interleukins in type 1 and type 2 diabetes. Abbreviations: IL, interleukins; T1D, type 1 diabetes; T2D, type 2 diabetes.

To synthesize current knowledge regarding the role of interleukins in diabetes, focusing on how these cytokines contribute to the disease’s pathophysiology and complications is crucial. Understanding these mechanisms may reveal new therapeutic avenues and inform clinical practice in managing diabetes. This approach aligns with the growing interest in targeting inflammatory pathways to improve outcomes in diabetes patients [13]. Therefore, the aim of this review paper was to explore the role of interleukins in the pathophysiology of diabetes forms, with an emphasis on recent developments, key research gaps, ongoing discussions, and potential future directions. Among the numerous interleukins, this review specifically highlights IL-1 through IL-35, discussing their pathophysiological roles in diabetes mellitus types, as illustrated in Table 1 and Figure 2.

## 2. Literature Search Strategy

This narrative review was conducted through a comprehensive search of the biomedical literature using electronic databases including PubMed, ScienceDirect, and Google Scholar. The search was finalized in January 2025 and included publications available up to that date. The following keywords and their combinations were used: “interleukins,” “cytokines,” “pathophysiology,” “type 1 diabetes,” “type 2 diabetes,” “immune regulation,” and “diabetes complications.”

To enhance the sensitivity of the search, reference lists of relevant articles were manually screened for additional eligible sources. Particular attention was given to both experimental and clinical studies investigating the role of interleukins in the onset, progression, and complications of diabetes mellitus.

Only peer-reviewed articles published in English were included in the review. While no strict publication date restriction was applied, studies published in the last 10 years were prioritized to ensure the inclusion of the most up-to-date and relevant data. Non-English publications were excluded due to limitations in translation resources and to avoid the misinterpretation of scientific findings.

## 3. Role of Interleukins on Diabetes Mellitus

### 3.1. Interleukin-1 (IL-1)

Interleukin-1 alpha (IL-1α) and interleukin-1 beta (IL-1β) are pro-inflammatory cytokines of approximately 17 kDa, produced by a wide range of immune and non-immune cells, including macrophages, monocytes, lymphocytes, neutrophils, fibroblasts, and epithelial cells. These cytokines act via IL-1 type I and II receptors, primarily modulating T cells, endothelial cells, and fibroblasts to drive inflammation, hematopoiesis, and differentiation of immune cells such as Th17 and IL-10-producing regulatory B cells. In contrast, IL-1 receptor antagonist (IL-1Ra), produced by similar cell types, competes for the same receptors without eliciting a signaling response, thus attenuating IL-1-mediated inflammation [49].

IL-1β plays a pivotal role in both type 1 and type 2 diabetes by mediating immune dysregulation, β-cell dysfunction, and chronic inflammatory responses. In T1D, IL-1β promotes Th1 and Th17 polarization and contributes to β-cell destruction by inducing nitric oxide production, leading to necrosis and the exposure of β-cell autoantigens [17,50]. This mechanism perpetuates autoimmunity and accelerates disease onset.

Clinical studies have shown elevated IL-1β levels in T1D patients, particularly in younger individuals and those with poor glycemic control, suggesting its potential as a biomarker of disease activity [14,15,16]. However, the efficacy of IL-1β is tightly regulated by IL-1Ra. An imbalance between these two molecules—via excess IL-1β or insufficient IL-1Ra—may shift the immune response toward chronic inflammation, as seen in both T1D and T2D [51,52]. Although the physiological role of IL-1Ra is acknowledged, the functional differences among its isoforms remain insufficiently understood, warranting further research [51]. In clinical trials, anakinra (recombinant IL-1Ra) has demonstrated improvement in glycemic control and β-cell preservation in T2D patients [53].

In T2D, IL-1β expression is upregulated by hyperglycemia through NF-κB pathway activation, resulting in β-cell apoptosis and impaired insulin secretion [18]. Ehses et al. further demonstrated IL-1β’s dual role in islet inflammation and systemic insulin resistance in Goto-Kakizaki rat models [10]. Studies also suggest that IL-1β inhibition preserves β-cell mass and improves metabolic outcomes, underlining its role in β-cell survival and function [54].

Genetic factors also influence IL-1β expression. The IL-1β C-511T promoter polymorphism has been linked to increased susceptibility to T2D and coronary artery disease, especially in CT or TT genotype carriers [55]. This suggests that IL-1β activity may be modulated by both genetic and environmental factors.

Beyond glycemic regulation, IL-1β contributes to the pathogenesis of diabetes complications. Elevated IL-1β levels have been associated with diabetic nephropathy, as it induces endothelial injury in renal tissues [56,57]. In diabetic retinopathy, IL-1β mediates the apoptosis of retinal capillary cells under hyperglycemic conditions [58]. These mechanisms underscore IL-1β’s broader role in chronic diabetic inflammation. Zhao et al. demonstrated via computational modeling that targeting IL-1β-driven inflammation is as critical as controlling hyperglycemia in halting T2D progression [59].

### 3.2. Interleukin-2 (IL-2)

Interleukin-2 (IL-2), a 15.5 kDa monomer, is secreted mainly by activated CD4^+^ and CD8^+^ T cells, as well as dendritic cells, NK cells, mast cells, NKT cells, and ILCs. Acting through the IL-2 receptor complex, IL-2 promotes the proliferation of effector T and B cells, supports the development of regulatory T cells, enhances NK cell function, and drives cytokine production in innate lymphoid cells [49].

IL-2 is a key cytokine that helps maintain the balance of the immune system. It plays a crucial role in supporting regulatory T cells (Tregs), which prevent the immune system from attacking the body’s own tissues. The IL2RA gene, which encodes the CD25 protein (a part of the high-affinity IL-2 receptor), is especially important in this process. Genetic changes in IL2 and IL2RA have been associated with T1D, particularly through the IDD3 locus on chromosome 4q27 [60]. Two studies explain how impaired IL-2 signaling contributes to T1D [60,61]. Normally, IL-2 helps Tregs suppress harmful immune responses. But when IL-2 or IL2RA function is disrupted, Tregs are unable to perform their function properly, leading to the autoimmune destruction of pancreatic β cells, suggesting that improving IL-2 signaling might help restore immune balance and slow disease progression [61]. Interestingly, another study reported that IL-2 levels were high not only in T1D children but also in their siblings. This suggests that increased IL-2 may reflect an early immune imbalance, potentially before the onset of T1D, supporting its role as a marker of immune dysregulation [19].

A major focus in recent research is the use of low-dose IL-2 (LD-IL-2) to treat T1D. This approach aims to selectively expand Tregs without activating harmful effector T cells (Teffs), offering more controlled immune modulation. Multiple studies confirm that IL-2 works in a dose-dependent manner, enhancing immune tolerance at lower doses [62,63]. However, some challenges exist. Anti-IL-2 autoantibodies, found in both humans with T1D and NOD mice, may interfere with IL-2 therapy, reducing its effectiveness [63]. This highlights the complexity of immune responses in autoimmune diseases and the need to monitor such antibodies during treatment. Novel approaches are expanding our knowledge of IL-2-based treatments. Zhang et al. [64], using multiomics analysis, showed that intermittent low-dose IL-2 (iLD-IL-2) expanded thymic-derived Tregs and decreased harmful IL-21-producing CD4+ cells. These effects lasted for a month, showing that IL-2 can provide long-lasting immune modulation. Similarly, Rosenzwajg et al. observed better β-cell preservation in children with strong Treg responses to IL-2 [65]. The ITAD trial evaluated ultra-low-dose IL-2 in pediatric T1D patients and found that it could slow disease progression. These findings support LD-IL-2 as a promising tool for maintaining immune tolerance, though long-term studies are still needed [66]. Innovations in IL-2 delivery and formulation are further enhancing its therapeutic potential. For example, Nagy et al. [67] used a hydrogel-based system to deliver IL-2 in NOD mice, making treatment more manageable and effective. IL-2/CD25 fusion proteins are another exciting advancement that improved IL-2’s stability and function in delaying diabetes in animal models [63]. Likewise, Qureshi et al. [68] supported CD25-targeted therapies for protecting β-cells in prediabetic models. However, not all effects are beneficial. Dong et al. [69] cautioned that even LD-IL-2 might unintentionally activate cytotoxic immune cells, which could worsen autoimmunity. Therefore, precise dose control is essential. Moreover, genetic differences in IL2RA may affect how individuals respond to IL-2 therapy, reinforcing the importance of personalized medicine [64].

Although IL-2 has been mainly studied in T1D, its role in T2D is emerging. Oncul et al. [70] found high IL-2 levels in rheumatoid arthritis patients with insulin resistance, suggesting a link between chronic inflammation and metabolic issues. Similarly, Suri et al. [20] observed anti-inflammatory IL-2 activity in newly diagnosed T2D patients, pointing to its potential as a diagnostic biomarker. More concerning is the possible connection between IL-2 and cancer risk. Bosek et al. [71] reported elevated IL-2 in T2D patients with colon cancer, implying that immune dysregulation may bridge diabetes and cancer. On the other hand, Ali et al. [72] showed that metformin therapy lowered IL-2 and TNF-α levels in people with diabetes, offering a way to reduce inflammation pharmacologically.

### 3.3. Interleukin-3 (IL-3)

Interleukin-3 (IL-3) is a monomeric cytokine of approximately 15 kDa, secreted by T cells, macrophages, natural killer (NK) cells, eosinophils, mast cells, and stromal cells. It signals through a receptor complex composed of IL-3Rα and a common β-chain (shared with GM-CSF and IL-5). IL-3 primarily targets hematopoietic progenitor cells, CD34^+^ stem cells, basophils, eosinophils, monocytes, regulatory T cells (Tregs), and endothelial cells. It plays a pivotal role in hematopoiesis by promoting the differentiation, survival, and activation of immune cells [49].

Recent studies have expanded our understanding of IL-3, suggesting that its role extends beyond hematopoiesis to immune modulation in inflammatory conditions. Depending on the disease context, inflammatory stage, and local immune environment, IL-3 may either exacerbate or resolve inflammation, reflecting its dual functional nature [73].

In type 1 diabetes (T1D), both genetic and experimental evidence support the involvement of IL-3 in immune regulation. Aged mice deficient in both IL-3 and granulocyte-macrophage colony-stimulating factors (GM-CSF) developed hallmark features of T1D, including insulitis, β-cell destruction, and impaired glucose tolerance. These mice also exhibited increased macrophage-derived p40 cytokines following lipopolysaccharide (LPS) stimulation. Interestingly, deletion of interferon-γ (IFN-γ) ameliorated these diabetic phenotypes, whereas the blockade of the inhibitory co-receptor CTLA-4 accelerated disease onset, underscoring IL-3’s role in maintaining immune balance [21].

In line with these findings, both T1D patients and non-obese diabetic (NOD) mice show blunted hematopoietic responses to IL-3 and GM-CSF, suggesting a shared defect in these signaling pathways. This dysfunction may impair regulatory mechanisms that are necessary for immune tolerance and β-cell preservation [21].

IL-3 also appears to directly support immunoregulatory cell populations. In NOD mice, exogenous IL-3 delayed diabetes onset and reduced disease incidence when administered during early life (2–4 weeks of age). Bone marrow cells harvested from IL-3-treated mice conferred protection against chemically induced diabetes when transferred to naïve recipients, though this effect was not observed in adoptive transfer models using autoreactive T cells, indicating a context-dependent immunomodulatory effect [74].

Further analysis revealed that IL-3 exposure promotes the generation of a distinct population of immature T cells (Thy-1^+^ CD3ε^lo^ CD4^−^ CD8^−^ CD25^−^) in the bone marrow, capable of delaying diabetes onset upon adoptive transfer. These cells, likely generated extrathymically, may represent a novel subset of regulatory immune cells [74].

Collectively, IL-3 demonstrates promising immunoregulatory properties, particularly in the early or preclinical stages of T1D. It fosters immune tolerance, supports regulatory immune cell populations, and may delay autoimmune progression, making it a potential candidate for preventive immunotherapy in individuals at high genetic risk.

However, the role of IL-3 in T2D remains poorly characterized. Unlike IL-1β, IL-6, or TNF-α, IL-3 has not been directly linked to the low-grade chronic inflammation associated with T2D. Its involvement in insulin resistance, adipose tissue inflammation, or β-cell stress has yet to be established, warranting further investigation into its relevance in metabolic disease contexts.

### 3.4. Interleukin-4 (IL-4)

Interleukin-4 (IL-4) is a 15 kDa monomeric cytokine primarily produced by Th2 cells, as well as basophils, eosinophils, mast cells, NKT cells, and γδ T cells. IL-4 signals through type I and type II IL-4 receptors and acts on T and B cells to promote Th2 differentiation, immunoglobulin class switching to IgE, and the upregulation of MHC class II and CD23 expression. These functions position IL-4 as a key mediator in anti-inflammatory responses, allergic immunity, and tissue homeostasis [49].

In T1D, a disease driven by Th1-mediated autoimmune destruction of pancreatic β-cells, IL-4 is considered a protective cytokine due to its capacity to counterbalance Th1-driven inflammation. Studies have shown that IL-4 production is impaired at disease onset. When PBMCs from newly diagnosed patients are stimulated in vitro, they exhibit significantly reduced IL-4 secretion compared to healthy controls. While some recovery of IL-4 levels is seen during the partial remission (“honeymoon”) phase, they remain suboptimal [22]. Similar impairments have been reported in NOD mice, a well-established T1D model [75].

Although IL-4 deficiency alone does not exacerbate insulitis severity in NOD mice, experimental strategies that enhance IL-4 expression, including systemic or local administration, or genetic engineering of β-cells and dendritic cells, can delay diabetes onset and reduce disease incidence. These effects are largely attributed to IL-4’s immunomodulatory actions, which promote regulatory T-cell expansion, suppress pro-inflammatory cytokine release, and protect β-cell function [76,77,78,79,80,81,82]. Collectively, these findings support IL-4’s potential as an early-stage immunotherapeutic agent in T1D.

T2D, although primarily metabolic in origin, is increasingly associated with chronic low-grade inflammation. Emerging evidence suggests that IL-4 may also be involved in this context. One clinical study found a positive correlation between serum IL-4 and IL-5 levels in T2D patients, suggesting a coordinated Th2 response. Interestingly, similar correlations were observed in healthy controls between IL-4, IL-5, and IFN-γ, indicating complex cross-regulation among Th1 and Th2 pathways [23].

While the precise role of IL-4 in T2D has yet to be fully elucidated, it may reflect an attempt by the immune system to mitigate pro-inflammatory signaling associated with insulin resistance. Alternatively, dysregulated Th2 responses could paradoxically exacerbate metabolic inflammation in susceptible individuals.

### 3.5. Interleukin-5 (IL-5)

Interleukin-5 (IL-5) is a 15 kDa homodimeric cytokine produced by Th2 cells, activated eosinophils, mast cells, TC2 cells, γδ T cells, NK/NKT cells, Peyer’s patch–derived innate cells, and type 2 innate lymphoid cells (ILC2s). IL-5 signals through the IL-5 receptor (IL-5Rα and βc chains), primarily targeting eosinophils, basophils, mast cells, Tregs, neutrophils, and monocytes. It regulates eosinophil proliferation, differentiation, and activation, and plays essential roles in allergic inflammation, wound healing, and tissue remodeling [49].

Although IL-5 is classically associated with allergic and eosinophilic responses, recent findings suggest a broader role in chronic inflammatory diseases such as T2D. In an immune profiling study, T2D patients exhibited a positive correlation between IL-5 and IL-4 levels, suggesting potential Th2-biased immune activation. Interestingly, in healthy controls, IFN-γ also correlated positively with both IL-4 and IL-5, indicating an intertwined Th1/Th2 cytokine network that may become dysregulated in the diabetic state [23].

Beyond systemic inflammation, IL-5 has been implicated in diabetes-related neurocognitive decline. One study found that elevated IL-5 levels in T2D patients were significantly associated with slower information processing speed in individuals with mild cognitive impairment (MCI), suggesting a link between IL-5 and neuroinflammation in metabolic disease [24]. This highlights IL-5’s potential relevance not only to immunometabolic dysfunction but also to neurological complications in T2D.

In contrast to T2D, the role of IL-5 in T1D remains less defined. Current evidence does not establish a direct link between IL-5 and β-cell autoimmunity. However, given its functional overlap with IL-4 and shared involvement in Th2 immunity, IL-5 may indirectly influence the immune milieu in T1D. Its interactions with B cells and eosinophils—both relevant to immune tolerance and inflammation—suggest that IL-5 could participate in modulating immune responses during disease onset or progression, though this remains speculative and requires further investigation [23].

### 3.6. Interleukin-6 (IL-6)

Interleukin-6 (IL-6) is a pleiotropic cytokine with a molecular weight between 19 and 26 kDa. It exerts its effects via binding to either the membrane-bound IL-6 receptor (IL-6R) or its soluble form (sIL-6R), together with the gp130 co-receptor. IL-6 is secreted by a wide range of cell types, including monocytes/macrophages, T and B lymphocytes, endothelial and epithelial cells, fibroblasts, adipocytes, smooth muscle cells, and glial cells. Its target cells include hepatocytes, leukocytes, lymphocytes, and hematopoietic progenitors. IL-6 mediates diverse biological processes such as acute-phase protein synthesis in the liver, leukocyte activation and migration, B-cell maturation, immunoglobulin production, T-cell differentiation, and regulation of hematopoiesis. It also contributes to bone resorption, angiogenesis, synovial proliferation, and cartilage degradation, and is involved in the neuroendocrine and stress responses via ACTH synthesis [49].

Although IL-6 was initially studied for its role in autoimmune inflammation, it is now recognized as a key player in metabolic regulation. IL-6 influences glucose and lipid metabolism in adipose tissue, skeletal muscle, liver, pancreatic β cells, and central neuroendocrine systems [26].

In T1D, IL-6 is not considered a primary pathogenic driver, but elevated IL-6 levels are frequently observed in patients. Polymorphisms in the IL-6 and IL6R genes have been associated with immune dysregulation, suggesting that IL-6 may contribute to T1D pathogenesis in conjunction with other cytokines such as IL-1β and TNF-α [25]. While these findings indicate a role for IL-6 in shaping the autoimmune microenvironment, the mechanistic details have yet to be fully elucidated.

In T2D, IL-6 plays a more well-defined and prominent role. Chronic low-grade systemic inflammation is a hallmark of T2D, and IL-6 levels are consistently elevated in individuals at risk or in the early stages of the disease. IL-6 promotes insulin resistance by disrupting insulin signaling pathways and enhancing the inflammatory milieu, particularly through the activation of the JAK/STAT and NF-κB pathways [26].

Prospective cohort studies have demonstrated that elevated IL-6 and C-reactive protein (CRP) levels are predictive of T2D onset, especially in middle-aged populations [83]. This highlights IL-6 as a potential early biomarker and therapeutic target in metabolic disorders. However, targeting IL-6 in clinical practice presents challenges. Due to its wide-ranging physiological roles, IL-6 blockades may impair host defense mechanisms and increase infection risk. Therefore, while IL-6 inhibition may offer therapeutic benefits, lifestyle modifications such as dietary changes and physical activity remain the cornerstone of T2D prevention and management [84].

### 3.7. Interleukin-7 (IL-7)

Interleukin-7 (IL-7) is a 25 kDa monomer that exerts its effects via the IL-7 receptor (IL-7R and soluble IL-7R). It is produced by epithelial cells, keratinocytes, dendritic cells, B cells, and monocytes/macrophages. IL-7 primarily targets developing B and T lymphocytes, mature T cells, NK cells, and innate lymphoid cells (ILCs). It plays a crucial role in early lymphopoiesis by promoting pre-B- and pro-B-cell proliferation (notably in mice), supporting megakaryocyte maturation, and facilitating V(D)J recombination. In mature immune cells, IL-7 ensures naïve T-cell survival, drives thymocyte expansion, and sustains ILC development and homeostasis. Moreover, it can induce the production of inflammatory mediators in monocytes [49].

IL-7 is a cytokine that plays a central role in the development and maintenance of T cells, which are key components of the immune system. Through its interaction with IL-7R, IL-7 helps naive CD4+ and CD8+ T cells mature into effector cells that produce IFN-γ, a pro-inflammatory molecule involved in autoimmunity [85,86]. At the same time, IL-7 reduces the expression of PD-1, an inhibitory receptor that normally helps prevent overactive immune responses. This reduction allows self-reactive T cells to become more aggressive, contributing to autoimmune damage in T1D [85,86].

However, IL-7’s function is not entirely harmful. It also supports the survival and function of regulatory T cells (Tregs), which are essential for maintaining immune tolerance and preventing autoimmune reactions [87]. Additionally, IL-7 can enhance the immunoregulatory function of dendritic cells (DCs), helping to dampen immune responses and potentially delay or prevent disease onset [88]. This dual role of IL-7 in both promoting inflammation and supporting immune regulation highlights the complex nature of its involvement in T1D.

Animal studies have helped to clarify this contradiction. In NOD mice, which are commonly used as a model for human T1D, IL-7 accelerates the development of diabetes, while blocking the IL-7R slows disease progression [85,86,89]. Blocking IL-7R reduces the number of inflammatory T cells (those that produce IFN-γ) and increases Treg numbers. Interestingly, therapy with antibodies against IL-7Rα has also been shown to increase PD-1 expression, essentially reactivating the body’s ability to regulate overly active immune cells [85]. These findings point to the IL-7/IL-7R pathway as a promising therapeutic target in T1D, especially for preserving insulin-producing β-cells. However, these promising results are mostly from animal models, and the effectiveness of this approach in humans still needs to be proven through clinical trials [6].

Human studies provide more insights into how IL-7 behaves during T1D. At the onset of T1D, higher levels of soluble IL-7 receptors (sIL-7R) have been observed in the blood, which later decrease following treatment. Meanwhile, actual IL-7 levels in the blood are typically higher in patients with long-standing T1D, suggesting a possible delayed effect or compensation mechanism in chronic disease [27]. Genetic studies have shown that there is a specific variant in the IL7RA gene (SNP rs6897932) and certain HLA types influence sIL-7R levels. Interestingly, these genetic factors do not seem to directly affect IL-7 concentrations. Instead, IL-7 levels appear to be closely tied to the levels of circulating sIL-7R, pointing to a complex interaction between the cytokine and its receptor system over time [27].

When it comes to T2D, there is currently little evidence that IL-7 plays a significant role. Unlike IL-6 or IL-1β, which are strongly linked to inflammation and insulin resistance in T2D, IL-7 has not been clearly associated with metabolic inflammation or glucose metabolism. This does not rule out a potential role, but it suggests that IL-7 is not a major player in T2D pathogenesis currently and warrants more investigation in this context.

### 3.8. Interleukin-8 (IL-8)

Interleukin-8 (IL-8), also known as CXCL8, is a pro-inflammatory chemokine with a molecular weight of approximately 16 kDa. It exerts its biological effects via CXCR1 and CXCR2 receptors and is produced by a wide range of cells including monocytes, macrophages, neutrophils, lymphocytes, endothelial and epithelial cells, fibroblasts, and hepatocytes. IL-8 primarily acts as a chemoattractant for neutrophils but also influences the recruitment and activation of T cells, NK cells, and other leukocytes. It plays additional roles in angiogenesis, hematopoietic stem cell mobilization, and vascular remodeling [49].

IL-8 has been implicated in both type 1 and type 2 diabetes, particularly in the context of chronic inflammation and its downstream complications. In T1D, elevated circulating IL-8 levels have been reported and may reflect the ongoing systemic inflammatory environment. For example, Syed Khaja et al. demonstrated that IL-8, along with IL-6 and IL-10, was significantly elevated in T1D patients—especially among smokers—suggesting that environmental factors such as tobacco use may amplify cytokine-mediated immune activation [28]. While these findings support the potential utility of IL-8 as a biomarker for disease activity or progression in T1D, its direct role in β-cell destruction remains uncertain.

In T2D, the involvement of IL-8 is more clearly established. Chronic low-grade inflammation is a hallmark of T2D, and IL-8 is consistently elevated in affected individuals. Studies have shown that IL-8 levels correlate with metabolic parameters such as glucose dysregulation, lipid abnormalities, and vitamin D deficiency, further supporting its role in metabolic derangement [90]. IL-8 has also been explored as a non-invasive salivary biomarker in T2D, although its correlation with glycemic control remains inconclusive [91].

Moreover, IL-8 contributes to the pathophysiology of diabetic nephropathy (DN). Its interaction with CXCR1/2 receptors on renal cells, particularly podocytes, has been shown to promote inflammation and injury, thereby facilitating the progression of kidney damage in diabetic patients [92]. In DN patients, a combined elevation of IL-8 and sTWEAK (soluble TNF-like weak inducer of apoptosis) has been suggested as a potential early diagnostic marker for renal complications [93].

While some studies have investigated IL-8 in the context of inflammatory comorbidities such as periodontitis or oral lichen planus in T2D patients, these associations may be better interpreted as reflections of systemic immune activation rather than primary mechanisms in diabetes pathogenesis [94,95]. Importantly, Borilova Linhartova et al. reported that IL-8 plasma levels were not significantly associated with CXCL8 or CXCR2 gene polymorphisms, indicating that environmental or epigenetic factors are likely stronger determinants of IL-8 expression in T2D [94].

Interestingly, IL-8 also shows variable behavior in endocrine conditions linked to insulin resistance. For instance, in female patients with subclinical hypothyroidism and obesity, IL-8 levels were inversely related to insulin resistance indices [96]. This suggests that IL-8’s role may be context-dependent, varying with hormonal and metabolic milieu.

In summary, IL-8 represents a multifaceted cytokine involved in the inflammatory, metabolic, and vascular complications of diabetes. Its potential as a diagnostic or prognostic biomarker is supported by growing clinical evidence, particularly in T2D and its renal sequelae. However, further research is needed to define its mechanistic contributions and therapeutic relevance across the spectrum of diabetic disorders.

### 3.9. Interleukin-9 (IL-9)

Interleukin-9 (IL-9) is a 14 kDa monomeric cytokine that signals via the IL-9 receptor (IL-9R), which is expressed on various target cells including B and T lymphocytes, mast cells, hematopoietic progenitors, airway and intestinal epithelial cells, and smooth muscle cells. IL-9 is primarily produced by Th2, Th9, Th17, regulatory T (Treg) cells, mast cells, eosinophils, and innate lymphoid cells (ILCs). It acts as a growth factor for T cells and mast cells, inhibits Th1 cytokine production, promotes CD8^+^ T cell and mast cell proliferation, and contributes to allergic inflammation by enhancing IgE, chemokine, and mucus secretion in airway epithelial cells [49].

Although historically overshadowed by Th1/Th2 cytokines in the context of T1D, recent research has begun to explore IL-9’s potential role in the immunopathogenesis and complications of diabetes. A cytokine profiling study assessing multiple immune mediators—such as IL-33, IL-12, IFN-γ, IL-4, IL-10, IL-17, and IL-9—in T1D patients with and without microvascular complications (MVCs) found that while cytokine levels were elevated in all diabetic patients compared to controls, there were no significant differences between those with and without MVCs. Notably, the cytokine profile did not clearly favor either a Th1 or Th2 polarization, suggesting a mixed or atypical immune response in T1D [97].

IL-9 has gained particular attention for its potential role in diabetic nephropathy (DN). A study involving young T1D patients found that urinary IL-9 levels correlated with markers of podocyte injury, specifically podocyte-derived extracellular vesicles (EVs). Podocytes are vital for glomerular filtration, and their damage marks the onset of diabetic kidney disease. IL-9 was also found to interact with pro-inflammatory mediators such as VEGF, TNF-α, and IL-6, supporting its involvement in renal inflammation. Importantly, IL-9 levels showed a positive correlation with the albumin-to-creatinine ratio (ACR), a key clinical indicator of renal dysfunction, suggesting IL-9 as a potential early biomarker of kidney stress in T1D [30].

Additional insights come from studies examining IL-9 levels across different stages of glucose metabolism, including individuals with normal glucose tolerance (NGT), diabetes, and diabetic kidney disease (DKD). These studies revealed a biphasic pattern: IL-9 levels were lower in individuals with diabetes compared to those with NGT but were significantly higher in patients with DKD. This observation suggests a possible dual role of IL-9—initially downregulated during early metabolic stress but upregulated in response to progressing renal injury. The increase in IL-9 in DKD also coincided with rising TGF-β levels and declining IL-17 expression, pointing to a potential compensatory or protective role in later stages of kidney disease [31].

Taken together, these findings indicate that IL-9 does not conform to traditional Th1/Th2 paradigms in diabetes. While it may not drive autoimmune β-cell destruction directly, its interactions with podocytes, pro-inflammatory cytokines, and renal function markers highlight its relevance in diabetic complications, particularly nephropathy. Elevated IL-9 in DKD may reflect an attempt to mitigate chronic inflammation and fibrosis, suggesting its potential as both a biomarker and therapeutic target in the later stages of diabetes-associated kidney disease.

### 3.10. Interleukin-10 (IL-10)

Interleukin-10 (IL-10) is a homodimeric cytokine with a predicted precursor size of 20.5 kDa and a mature monomeric form of approximately 18.6 kDa. It signals through a receptor complex composed of IL-10R1 and IL-10R2. IL-10 is secreted by various immune cells, including T cells, B cells, monocytes, macrophages, and dendritic cells (DCs). It exerts its immunoregulatory effects on macrophages, monocytes, T cells, B cells, NK cells, mast cells, DCs, and granulocytes. Functionally, IL-10 suppresses inflammatory responses by inhibiting the activity of antigen-presenting cells and modulating T-cell differentiation. It also promotes IgG production while inhibiting IgE synthesis in B cells [49].

IL-10 is widely recognized as a key anti-inflammatory cytokine that maintains immune homeostasis and protects tissues during chronic inflammation. Beyond its classical role in immune cells, IL-10 also acts on neurons and adipocytes, contributing to tissue repair and modulating T cell activation, especially among CD8^+^ populations [8]. These diverse functions have made IL-10 a central molecule in understanding the immunopathology of both T1D and T2D.

In T1D, IL-10 levels are often elevated, particularly in patients presenting with diabetic ketoacidosis (DKA). This elevation correlates positively with HbA1c levels and inversely with age at disease onset, suggesting that IL-10 may reflect heightened immune activation, particularly in younger individuals [32]. Notably, IL-10 is often co-elevated with pro-inflammatory cytokines such as TNF-α, implying a compensatory upregulation in response to systemic inflammation [32].

Environmental and clinical modifiers also influence IL-10 dynamics in T1D. For instance, decreased IL-10 in T1D patients with oral pathologies suggests impaired mucosal immunity under hyperglycemic conditions [98]. Additionally, factors like vitamin D deficiency and smoking have been associated with increased IL-10 levels, further demonstrating the cytokine’s sensitivity to extrinsic influences [28].

IL-10 may also serve as a predictive biomarker. One longitudinal study reported age-related increases in IL-10, IL-1α, and IL-1β, potentially contributing to chronic low-grade inflammation preceding T1D onset [17]. Furthermore, IL-10 plays a protective role in bone metabolism; mice lacking IL-10 exhibited enhanced bone loss and reduced osteoblast function under hyperglycemic stress, highlighting IL-10’s relevance in diabetes-associated bone complications [99].

Its regulatory capacity is further supported by findings from BDC2.5^+^ NOD mice, in which IL-10 deficiency led to worsened insulitis and disrupted immune tolerance, likely due to altered neutrophil and CD4^+^ T-cell dynamics. Gut microbiota were also implicated in these changes, emphasizing the IL-10–microbiome–immunity axis in diabetes pathogenesis [100].

In T2D, IL-10 has garnered interest for its genetic and functional implications. One of the most extensively studied variants, rs1800896 (−1082 G/A), is located in the IL-10 gene promoter and influences transcriptional activity. Carriers of the A allele often exhibit reduced IL-10 production, leading to diminished anti-inflammatory signaling. This reduction is thought to exacerbate insulin resistance by allowing the unchecked activity of pro-inflammatory cytokines such as TNF-α and IL-6, which impair insulin receptor signaling through pathways like NF-κB and JNK. Consequently, individuals with this genotype may be more vulnerable to systemic inflammation and metabolic dysfunction [33]. Conversely, other polymorphisms such as −819 C/T, −592 A/C, and −1082 A/G have been associated with protection against diabetic nephropathy in specific populations, although these associations require further validation [98].

Finally, IL-10 plays a role in diabetic neuropathy. In db/db mice, a model of T2D, reduced neuronal IL-10 was linked to heightened pain sensitivity and neuroinflammation. Enhancing IL-10 signaling in this context has been proposed as a strategy to alleviate painful diabetic neuropathy (PDN), highlighting the cytokine’s potential in managing chronic complications [101].

### 3.11. Interleukin-12 (IL-12)

Interleukin-12 (IL-12) is a heterodimeric cytokine composed of p35 and p40 subunits, primarily secreted by monocytes, macrophages, dendritic cells, neutrophils, and B cells. It signals through the IL-12 receptor complex (IL-12Rβ1 and IL-12Rβ2) and plays a pivotal role in the differentiation and maintenance of T helper 1 (Th1) cells, the activation of natural killer (NK) cells, and the promotion of cytotoxic immune responses [49].

IL-12 has long been implicated in the pathogenesis of type 1 diabetes (T1D), particularly through its ability to enhance Th1-mediated autoimmunity. In non-obese diabetic (NOD) mice, IL-12 accelerates disease onset by increasing IFN-γ secretion and autoreactive T-cell infiltration into pancreatic islets [102]. Moreover, recent studies have shown that pancreatic β cells themselves can express both IL-12 and its receptors under inflammatory conditions, resulting in impaired glucose-stimulated insulin secretion and the upregulation of IFN-γ, which can be reversed by IL-12 neutralization [103]. These findings indicate that IL-12 may contribute directly to β-cell dysfunction, beyond its immunomodulatory effects.

Genetic studies support IL-12’s involvement in diabetes susceptibility. Polymorphisms such as the IL-12p40 1159A allele have been associated with increased gene expression and higher risk of T1D [34]. Additionally, IL-12 may have dual roles depending on context; while generally pro-inflammatory, intermittent low-dose IL-12 administration has been shown to reduce Th17-mediated autoimmunity under certain conditions in NOD mice [104].

In T2D, IL-12 is increasingly recognized for its role in chronic inflammation, insulin resistance, and vascular complications. Hyperglycemia can induce IL-12 production via PKC, JNK, and MAPK pathways, contributing to systemic inflammation and endothelial dysfunction [105]. Animal studies demonstrate that IL-12 deficiency enhances vascular repair, promotes angiogenesis, and reduces oxidative stress in diabetic models, even in the presence of obesity and insulin resistance [106,107]. These findings underscore IL-12’s pathogenic role in impaired wound healing, diabetic retinopathy, neuropathy, and atherosclerosis.

A recent review further emphasizes that IL-12 family cytokines (including IL-23 and IL-27) are deeply involved in diabetic complications, modulating pathways associated with neuroinflammation, angiopathy, and metabolic stress [108]. Blocking IL-12 signaling has shown promising results in reducing endothelial damage, restoring autophagic balance, and improving glucose homeostasis, suggesting therapeutic potential beyond glycemic control alone.

Furthermore, elevated IL-12 levels have also been linked to insulin resistance in other inflammatory conditions, such as acne vulgaris, pointing to a broader role in immunometabolic disorders [35]. Collectively, these findings identify IL-12 as a central mediator of both autoimmune and metabolic pathways in diabetes and its complications, highlighting its potential as a therapeutic target.

### 3.12. Interleukin-17 (IL-17)

Interleukin-17 (IL-17), particularly IL-17A, is a pro-inflammatory cytokine predominantly produced by Th17 cells and is increasingly recognized for its involvement in the immunopathogenesis of both type 1 and type 2 diabetes. IL-17 mediates its effects through the IL-17RA/RC receptor complex and activates downstream NF-κB and JNK pathways, leading to the production of pro-inflammatory cytokines, chemokines, and the recruitment of neutrophils, thereby amplifying tissue inflammation [109].

In type 1 diabetes (T1D), IL-17 contributes to pancreatic β-cell dysfunction and apoptosis. Animal models such as Akita and NOD mice have shown that deletion or inhibition of IL-17A improves glycemic control, reduces β-cell damage, and suppresses islet inflammation [36,110]. Clinical studies support these findings, demonstrating elevated IL-17 levels in newly diagnosed T1D patients and within pancreatic islets, not only from infiltrating immune cells but also from β and α cells under metabolic stress [111]. Moreover, genetic variants such as the IL-17A −197 A allele have been associated with increased IL-17 production, poor glycemic control, and heightened risk of comorbidities such as periodontitis in T1D [112].

In T2D, IL-17 has emerged as a key mediator linking chronic inflammation with metabolic dysregulation. Elevated IL-17 levels are associated with insulin resistance, hyperglycemia, dyslipidemia, and increased risk of complications such as diabetic nephropathy and retinopathy [37,113]. IL-17 interferes with insulin signaling pathways and exacerbates β-cell stress, while its inhibition improves glucose uptake in skeletal muscle and enhances insulin sensitivity in diabetic animal models [114]. Telmisartan, for example, has been shown to exert beneficial metabolic effects by downregulating IL-17 [114].

The role of IL-17 extends to diabetic complications. It promotes endothelial dysfunction, accelerates kidney inflammation and fibrosis, and may aggravate vascular injury when acting synergistically with hyperglycemia [115]. Additionally, IL-17-driven immune imbalance, particularly an elevated Th17/Treg ratio, has been implicated in worsening systemic inflammation and disease progression [116].

Given this broad pathogenic profile, IL-17 has gained attention as a therapeutic target. Anti-IL-17 monoclonal antibodies and Th17 inhibitors have shown promise in preclinical models by preserving β-cell integrity and mitigating metabolic complications [117,118]. Notably, Abdel-Moneim et al. emphasized IL-17’s dual contribution to inflammation and insulin resistance, suggesting that its blockade may benefit both autoimmune and metabolic aspects of diabetes [117].

### 3.13. Interleukin-18 (IL-18)

Interleukin-18 (IL-18) is a 22.3 kDa pro-inflammatory cytokine belonging to the IL-1 family. It is secreted by various immune and non-immune cells, including macrophages, dendritic cells, epithelial cells, chondrocytes, osteoblasts, renal tubular epithelial cells, and Kupffer cells. IL-18 acts through its specific receptor and synergizes with IL-12 to stimulate IFN-γ production, enhance NK cell cytotoxicity, and promote T helper cell polarization toward Th1 or Th2 responses depending on the cytokine environment [49].

IL-18 has been identified as a key immunometabolic mediator in the pathogenesis of both type 1 and type 2 diabetes. In T1D, elevated IL-18 levels have been consistently reported in both pediatric and adult patients and are associated with disease onset and progression. Harms et al. found significantly increased IL-18 expression in the blood and pancreatic tissues of T1D patients, with insufficient compensatory elevation in natural inhibitors such as IL-18BP and IL-37, leading to unregulated pro-inflammatory signaling [119]. Similarly, Altinova et al. observed that IL-18 levels positively correlated with HbA1c and postprandial glucose levels, and negatively with HDL cholesterol, reinforcing its role in metabolic dysregulation [38]. However, its association with microvascular complications was limited, suggesting that IL-18 primarily reflects active inflammation rather than long-term structural damage.

Genetic studies further implicate IL-18 in T1D susceptibility. The −137 GC promoter polymorphism has been linked to increased IL-18 expression and a more robust Th1 immune response, which plays a pivotal role in β-cell destruction [120]. Supporting this, AMNEL et al. reported elevated IL-18 in a high-risk sibling with multiple autoantibodies who was later diagnosed with T1D, suggesting that IL-18 may rise in preclinical stages [121]. Animal studies reinforce this pathogenic role—blocking IL-18 with IL-18 binding protein (IL-18BP) in streptozotocin-induced diabetic mice significantly reduced islet inflammation, nitric oxide, IFN-γ, and IL-1β levels, demonstrating that IL-18 initiates β-cell damage via inflammatory signaling [122].

Beyond autoimmunity, IL-18 plays a central role in the progression of diabetes-related complications, especially diabetic nephropathy. Chronic IL-18-driven inflammation promotes endothelial injury, vascular remodeling, and kidney dysfunction [123]. A recent Mendelian randomization study provided compelling evidence that genetically elevated IL-18 levels are causally linked to an increased risk of T2D [124]. This strengthens the argument that IL-18 is not merely a marker of inflammation, but an active contributor to β-cell failure, insulin resistance, and long-term diabetic complications.

In T2D, elevated IL-18 has been associated with worse lipid profiles, hyperglycemia, and increased inflammatory markers such as IL-6 and high-sensitivity CRP [125]. Longitudinal data from Thorand et al. showed that IL-18 is a strong predictor of T2D incidence, especially when combined with elevated IL-6 or CRP levels [126]. Interestingly, despite elevated IL-18 concentrations, some T2D patients exhibit impaired IL-18 responsiveness—termed “IL-18 resistance” due to the downregulation of IL-18 receptors on immune cells. This leads to reduced IFN-γ production and weakened inflammatory control, analogous to insulin resistance [127].

However, IL-18’s role as a clinical biomarker is still debated. While some studies show its potential for predicting T2D, others (e.g., Younus et al.) found no significant differences in IL-18 levels between T2D patients and controls, nor utility in diagnostic prediction using ROC analysis [128]. These discrepancies highlight the need to consider population-specific factors and disease stage when interpreting IL-18’s diagnostic relevance.

Altogether, IL-18 represents a critical node in the inflammatory network underlying diabetes. It contributes to β-cell dysfunction, promotes insulin resistance, and exacerbates complications like nephropathy. Future studies are warranted to explore IL-18-targeted therapies as potential strategies to mitigate both the immunologic and metabolic burden of diabetes.

### 3.14. Interleukin-23 (IL-23)

Interleukin-23 (IL-23) is a heterodimeric cytokine composed of p19 and p40 subunits and signals through the IL-23 receptor. It is primarily produced by phagocytic cells such as macrophages and activated dendritic cells, acting on Th17 cells, NK and NKT cells, eosinophils, monocytes, and epithelial cells. IL-23 plays a critical role in promoting IL-17 production, T-cell proliferation, memory development, and innate immune activation [49].

Recent research has identified IL-23 as a key amplifier of inflammation in both type 1 and type 2 diabetes. In T1D, IL-23 exacerbates pancreatic inflammation and accelerates β-cell destruction by enhancing the production of cytokines such as IL-17, IFN-γ, TNF-α, and IL-18. Notably, this effect appears to be phase-dependent—IL-23 promotes disease progression only when administered during β-cell damage, but not prior to it, indicating its role as a disease enhancer rather than an initiator [129].

Clinical studies have also demonstrated elevated IL-23 levels in children with newly diagnosed T1D, particularly in the presence of reduced IL-2 and increased IL-21 concentrations. This cytokine imbalance was associated with unfavorable lipid profiles, suggesting a link between immune dysregulation and metabolic dysfunction [40]. While IL-21 and IL-23 promote pro-inflammatory responses, IL-2 seems to exert a regulatory or protective role in T1D pathogenesis.

However, findings across human studies are not entirely consistent. Several reports found no significant differences in IL-23 levels between T1D patients and healthy controls, suggesting that IL-23 activity may vary based on disease stage or immunogenetic background [130,131]. Supporting this, IL23A gene variants, such as the GG haplotype, were associated with protection against T1D, whereas IL23R polymorphisms and serum IL-23 levels showed no definitive associations [130].

In T2D, IL-23 has similarly been implicated in maintaining a state of chronic low-grade inflammation. Elevated IL-23 levels have been reported in untreated T2D patients and obese women with diabetes, reinforcing its role in systemic immune activation [41,42]. Interestingly, antidiabetic therapies such as sitagliptin were shown to reduce IL-23 expression and downstream signaling molecules (JAK2, STAT3, RORγt), while upregulating negative regulators like SOCS1/3, indicating that therapeutic modulation of the IL-23 axis may have anti-inflammatory benefits [132].

The pathogenic role of IL-23 is further underscored by its interactions within the IL-23/Th17 axis, which promotes IL-17 production, tissue inflammation, and autoimmunity. This axis is not only central to T1D but is also well-established in other autoimmune diseases such as psoriasis and inflammatory bowel disease (IBD), where IL-23 inhibitors have shown therapeutic success [130,133].

Several anti-inflammatory cytokines have emerged as potential modulators of IL-23-driven inflammation. IL-35, a potent immunosuppressive cytokine, is typically reduced in T2D patients, with levels improving after treatment [41]. In animal models of T1D, IL-35 therapies reduced inflammation, preserved β-cell function, and promoted a shift in macrophage polarization from pro-inflammatory (M1) to anti-inflammatory (M2) phenotypes [129].

Similarly, low-dose IL-2 therapy, particularly in nanoparticle formulations, was shown to improve early-stage T1D by suppressing IL-23 expression and maintaining IL-25 levels, suggesting an immunoregulatory effect that may involve balancing effector and regulatory pathways [40]. TGF-β, another anti-inflammatory cytokine, was found to be significantly reduced in T1D patients, whereas IL-17 and IL-23 levels remained unchanged in some studies, further highlighting TGF-β’s possible protective function [134].

Lastly, demographic factors such as age and sex may influence IL-23-related immune responses. Older patients and women with T2D were shown to have higher levels of oxidative stress and pro-inflammatory cytokines, including IL-23, indicating the need for personalized, sex- and age-sensitive approaches in diabetes management [135].

### 3.15. Interleukin-27 (IL-27)

Interleukin-27 (IL-27) is a heterodimeric cytokine composed of the p28 and EBI3 subunits and signals through a receptor complex formed by WSX-1 and gp130. It is produced primarily by activated dendritic cells, macrophages, and epithelial cells. IL-27 acts on T cells and NK cells, promoting T-bet expression and Th1 differentiation via STAT1 signaling while concurrently suppressing Th17 responses [49].

IL-27 has gained increasing attention in diabetes research due to its complex immunomodulatory functions. Evidence from experimental and clinical studies suggests that IL-27 plays a dual role in the pathogenesis of both type 1 and type 2 diabetes, with its effect being dependent on disease context, the immune environment, and disease stage.

In autoimmune diabetes models, IL-27 generally exhibits a pathogenic role. In NOD mice, genetic deletion of IL-27 or its receptor conferred complete protection from T1D and significantly reduced insulitis. IL-27 derived from myeloid cells disrupted immune balance by reducing Tregs and promoting Th1 and cytotoxic CD8^+^ T-cell responses, ultimately accelerating β-cell destruction [136,137].

Elevated IL-27 levels in diabetic mice have been linked to increased production of pro-inflammatory cytokines such as IFN-γ and IL-17 and reduced levels of anti-inflammatory mediators including IL-10 and TGF-β, further reinforcing a pro-autoimmune environment [137]. In human studies, myeloid dendritic cells from T1D patients displayed upregulated IL-27 receptor expression and enhanced downstream signaling, along with elevated expression of checkpoint inhibitors like PD-L1 and PD-1, suggesting active involvement in immune regulation during disease progression [43].

Interestingly, Helicobacter pylori infection—particularly with the virulent CagA-positive strain—has been shown to suppress IL-27 production in T1D patients, pointing to a potential interaction between microbial exposure and cytokine regulation in diabetes [138].

Despite this evidence, IL-27 may exert protective effects in specific contexts. In streptozotocin (STZ)-induced models of diabetes, mice deficient in IL-27 signaling components (EBI3^−^/^−^ or WSX-1^−^/^−^) developed more severe hyperglycemia and islet inflammation. Treatment with recombinant IL-27 ameliorated glucose levels, improved proinsulin secretion, and reduced immune cell infiltration, suggesting a protective anti-inflammatory role in chemically induced diabetes [139]. These contrasting effects likely reflect differences between autoimmune and toxin-mediated disease models, the timing of cytokine exposure, or immune cell dynamics, indicating that IL-27 may be context-dependent in its actions.

From a genetic standpoint, IL-27 polymorphisms do not appear to significantly influence T1D susceptibility. A study in a Brazilian cohort reported no functional or regulatory differences in IL-27 gene variants between patients and controls, suggesting that IL-27-related risk may be modulated by environmental or post-translational factors rather than by inherited variants [140].

In T2D, the role of IL-27 is less clearly defined but appears to differ by disease stage. In newly diagnosed T2D patients, IL-27 secretion in response to toll-like receptor (TLR) stimulation was reduced, indicating the early impairment of innate immune regulation. In contrast, long-standing T2D was characterized by increased T-cell activation and chronic inflammation, which may reflect compensatory immune activation following earlier cytokine insufficiency [141].

Moreover, in obesity and metabolic syndrome, IL-27 has been shown to exert anti-inflammatory effects and improve glucose and lipid metabolism. Preclinical data suggest that IL-27 may help modulate insulin sensitivity and reduce systemic inflammation. Recent reviews propose IL-27 as a potential therapeutic target, although its pleiotropic immune effects warrant further investigation before clinical application [44,142].

### 3.16. Interleukin-33 (IL-33)

Interleukin-33 (IL-33) is a 30 kDa cytokine (active form ~18 kDa) with a β-trefoil fold structure that signals through the ST2 receptor. It is released upon necrotic cell death and produced by nuocytes, fibroblasts under mechanical stress, and stromal or epithelial cells following tissue injury. IL-33 exerts its biological effects on a broad spectrum of immune and non-immune cells including basophils, mast cells, eosinophils, dendritic cells (DCs), macrophages, NK and NKT cells, T and B lymphocytes, endothelial and epithelial cells, fibroblasts, and innate lymphoid cells (ILCs). Functionally, IL-33 promotes TH2-type responses at mucosal barriers, supports dendritic cell maturation and cytokine release, enhances integrin expression on granulocytes, and activates ILCs [49].

IL-33 has emerged as a pleiotropic cytokine with both protective and pathogenic roles in diabetes, depending on the disease type, tissue microenvironment, and stage of disease progression. In T1D, several experimental studies suggest a protective role for IL-33. Lin et al. demonstrated that IL-33 improves pancreatic β-cell survival under diabetogenic stress by enhancing insulin secretion and mitochondrial function through the activation of the PPARγ signaling pathway, which increases energy production and reduces oxidative stress [143]. Similarly, studies by Pavlovic et al. and Lu et al. found that IL-33 promoted the expansion of Tregs, leading to decreased pancreatic inflammation, reduced β-cell apoptosis, and delayed or prevented T1D onset in murine models [144,145]. Ryba-Stanisławowska et al. reported that IL-33 enhanced FOXP3 expression and increased Treg populations in T1D patients, indicating its potential in restoring immune tolerance [45].

However, the role of IL-33 is more complex in diabetic complications, particularly in DKD. Hofherr et al. found elevated renal IL-33 expression in DKD patients and demonstrated that the inhibition of IL-33 signaling alleviated kidney inflammation and fibrosis in diabetic mice, suggesting a pathogenic role in this context. These findings are being further explored in the clinical trial FRONTIER-1, which evaluates IL-33-targeting monoclonal antibodies for the treatment of DKD [146].

In T2D, IL-33 shows context-dependent effects. Singh et al. reported lower circulating IL-33 and higher levels of soluble ST2 (sST2) in T2D patients compared to healthy individuals, with IL-33 negatively correlating with glycemia, implying a protective metabolic role [46]. Conversely, Pereira et al. observed increased IL-33 expression in the subcutaneous adipose tissue of T2D patients, where it was associated with impaired glucose uptake via the downregulation of GLUT4 and metabolic genes, linking IL-33 to insulin resistance in adipocytes [147]. Lin et al.’s earlier findings regarding IL-33’s protective role in pancreatic islets through the PPARγ axis [143] underscore the tissue-specific effects of this cytokine.

Systematic analysis by Missous et al. revealed inconsistent findings regarding IL-33 levels in diabetes, noting high inter-individual variability and emphasizing the need for standardized detection methods [148]. In addition, Shelest et al. associated elevated IL-33 levels with a higher incidence of pulmonary hypertension (PH) in T2D patients with coronary artery disease, proposing IL-33 as a possible biomarker for cardiovascular complications [149].

These observations underscore the duality of IL-33 in diabetes pathogenesis and complications—offering protection through immune regulation and β-cell preservation in some contexts, while promoting inflammation and metabolic dysfunction in others. As suggested by Liew et al. in their comprehensive review, IL-33’s function appears to be highly context-dependent, requiring further research to resolve its paradoxical roles and potential therapeutic value in both T1D and T2D [144].

### 3.17. Interleukin-35 (IL-35)

Interleukin-35 (IL-35) is a 60 kDa heterodimeric cytokine composed of the p35 and EBI3 subunits, signaling through various receptor complexes including IL-12Rβ2/gp130, IL-12Rβ2 homodimers, and gp130 homodimers. It is primarily secreted by regulatory T cells (Tregs), as well as by monocytes, vascular endothelial cells, smooth muscle cells, and epithelial cells. IL-35 exerts strong immunosuppressive effects by inhibiting effector T-cell proliferation, enhancing IL-10 secretion, and expanding the Treg population [49].

As a member of the IL-12 cytokine family, IL-35 has gained attention for its anti-inflammatory and immunoregulatory functions, particularly in the context of T1D. Recent studies emphasize its protective role in controlling autoimmune responses by modulating key immune cell populations. Notably, reduced IL-35 levels have been associated with the onset and severity of T1D, while IL-35-based therapies have shown promise in mitigating disease progression.

IL-35 functions by suppressing pro-inflammatory T-cell subsets, such as Th1 and Th17 cells, and by promoting the development and activity of regulatory cells, including Tregs and regulatory B cells (Bregs) [46,150,151]. It can also induce a specialized subset of Tregs known as iTr35, which further contributes to the suppression of immune-mediated inflammation [151].

In experimental models of T1D, including multiple low-dose streptozotocin (MLDSTZ) and NOD mice, administration of IL-35 either prevented or reversed the onset of diabetes. Treatment restored the function of previously impaired Tregs and shifted the immune environment toward an anti-inflammatory state [46].

Clinical studies support these findings. Lower IL-35 levels have been linked with increased β-cell destruction and more advanced disease. For instance, Chakraborty et al. and Bala et al. reported that patients with absent C-peptide secretion—a marker of diminished endogenous insulin production—exhibited significantly lower IL-35 levels and reduced numbers of IL-35-producing cells, including Tregs, Bregs, and CD8^+^FoxP3^+^ cells [47]. In contrast, Espes et al. observed that long-term T1D patients who retained some β-cell function had higher circulating IL-35 and a greater abundance of IL-35^+^ regulatory cells, alongside lower levels of pathogenic IL-17a^+^ cells [152].

Bregs also serve as a key source of IL-35 and play an important role in maintaining immune tolerance. Luo et al. demonstrated that diabetic mice had fewer IL-35^+^ Bregs, but IL-35 treatment increased their numbers and suppressed IFN-γ–producing inflammatory B cells, indicating that IL-35 supports both Breg and Treg-mediated regulation [153].

Additionally, IL-35 influences macrophage polarization, a critical component of pancreatic inflammation. In T1D, a shift toward M1-type (pro-inflammatory) macrophages exacerbates tissue damage. IL-35 administration has been shown to reduce the M1/M2 ratio, decrease blood glucose levels, and promote M2-like (anti-inflammatory) macrophages, suggesting an immunomodulatory role early in disease development [153].

Beyond its effects on glucose metabolism and β-cell preservation, IL-35 may also alleviate diabetes-related complications. For example, Jiang et al. reported that IL-35 reduced diabetic neuropathic pain by decreasing spinal cord inflammation and neuronal apoptosis, potentially through the inhibition of the JNK signaling pathway, which is involved in pain processing and inflammation [48].

Together, these findings underscore IL-35’s multifaceted role in T1D by modulating both innate and adaptive immune responses, preserving β-cell function, and offering a potential therapeutic benefit in managing both disease onset and complications.

### 3.18. Innate Immune Cells in Pathogenesis and Modulation of Type 1 Diabetes

Cell–cell crosstalk within the innate immune system plays a fundamental role in the pathogenesis and progression of T1D, influencing immune activation, β-cell destruction, and tolerance mechanisms. Various innate immune cells—including dendritic cells (DCs), macrophages, neutrophils, natural killer cells, and innate lymphoid cells—contribute to both disease initiation and regulation.

Dendritic cells (DCs) act as central antigen-presenting cells in T1D, capturing β-cell autoantigens and presenting them to autoreactive T cells in pancreatic lymph nodes, thereby initiating adaptive immune responses. However, tolerogenic DCs can induce Tregs, promoting immune tolerance and protecting against disease progression [136].

Macrophages are among the first immune cells to infiltrate pancreatic islets during insulitis. Pro-inflammatory M1 macrophages secrete IL-1β, TNF-α, and nitric oxide, directly contributing to β-cell apoptosis. In contrast, M2 macrophages, characterized by IL-10 and TGF-β production, may support tissue repair and limit autoimmunity [49].

Neutrophils also participate in the early phases of T1D, producing reactive oxygen species (ROS) and releasing neutrophil extracellular traps (NETs), which may serve as endogenous danger signals that promote autoimmune activation [43]. Natural killer cells contribute to β-cell destruction via cytotoxic granule release and by shaping adaptive immunity. While some studies associate NK cell activation with accelerated insulitis, others report regulatory functions under specific cytokine milieus [43]. ILCs, particularly ILC2s, have recently been implicated in immune regulation in diabetes. IL-33-responsive ILC2s secrete amphiregulin and IL-13, which have been shown to enhance β-cell survival, suppress inflammation, and promote glucose homeostasis in experimental models [45].

In summary, innate immune cell crosstalk plays a pivotal role in shaping the pancreatic microenvironment in T1D. While many of these cells contribute to disease initiation and progression, they also offer opportunities for immune modulation and prevention, depending on the surrounding cytokine context, receptor engagement, and timing. Strategies aiming to shift this balance toward tolerance rather than inflammation—such as tolerogenic DCs, M2 macrophages, or ILC2-based therapies—hold promise for future immunomodulatory interventions in autoimmune diabetes.

## 4. Methodological Limitations and Evidence Strength

This narrative review provides an integrative overview of the roles of interleukins in the pathogenesis of both type 1 and type 2 diabetes; however, several methodological and translational limitations should be acknowledged. As a narrative review, this work lacks the structured methodology of systematic reviews; specifically, it does not include predefined inclusion/exclusion criteria, formal risk-of-bias assessments, or quantitative synthesis through meta-analysis. As a result, the strength and generalizability of the conclusions are inherently dependent on the methodological rigor and reproducibility of the individual studies cited.

A significant proportion of the included evidence derives from preclinical studies, including in vitro experiments and animal models (e.g., NOD mice), which—while invaluable for elucidating mechanistic pathways—may not fully replicate the complexity of human immune-metabolic interactions. Species-specific immune responses, genetic background differences, and environmental exposures pose important barriers to direct translational application. While efforts were made to include human studies wherever available, the clinical data are limited in both scope and sample size. Many studies lack a longitudinal design, comprehensive adjustment for confounding variables (e.g., obesity, medication use, comorbidities), and external validation, which limits the robustness of observed associations.

Furthermore, publication bias likely affects the current body of the literature, as studies reporting positive or significant associations between interleukins and diabetes pathogenesis are more readily published than those with null findings. Heterogeneity in study populations, cytokine assay methodologies, sampling timing, and inter-individual variability in immune responses further complicate cross-study comparisons and the interpretation of cytokine levels.

Despite these challenges, the present review is grounded in peer-reviewed, biologically plausible evidence drawn from multiple major databases and reflects converging data from experimental and clinical studies. Nevertheless, to translate these findings into clinical practice, future investigations must prioritize well-designed longitudinal cohort studies, interventional trials targeting specific interleukin pathways, and systematic reviews with a standardized methodology to evaluate the consistency, effect size, and causality of interleukin-mediated mechanisms in diabetes.

## 5. Clinical Implications and Practical Utility of Interleukins

The expanding understanding of interleukin (IL) biology in diabetes mellitus has significant translational relevance, offering new avenues for biomarker development and targeted therapy. In T1D, interleukins such as IL-1β, IL-2, IL-6, and IL-8 are involved in the immunopathogenesis of β-cell destruction and autoimmune progression. Among these, low-dose IL-2 (LD-IL-2) therapy has shown promise in selectively expanding regulatory T cells (Tregs), enhancing immune tolerance, and preserving residual β-cell function in recent-onset T1D patients [64,65,66].

IL-1β, a potent pro-inflammatory cytokine, has been targeted in clinical trials with IL-1 receptor antagonists like anakinra, demonstrating improved glycemic control and β-cell preservation in T2D patients [53,54]. Similarly, IL-6, while pleiotropic, has emerged as a therapeutic candidate due to its consistent association with insulin resistance and cardiovascular risk in T2D [25,26,83,84].

From a diagnostic perspective, several interleukins have potential as accessible and non-invasive biomarkers. IL-6 and IL-8 levels have been independently associated with metabolic deterioration and cardiovascular risk in T2D [83,90]. Urinary IL-9 has been identified as a potential early indicator of diabetic nephropathy in T1D youth [30], while elevated IL-5 has been linked to cognitive impairment in patients with T2D [24].

Moreover, salivary IL-8 levels offer practical utility in non-invasive screening for T2D and its complications [91]. These findings suggest that interleukin profiling could complement traditional markers such as HbA1c and CRP in risk stratification and disease monitoring.

Despite these advances, the routine clinical application of interleukin-based diagnostics or therapies remains limited. Variability in assay methodologies, a lack of universally accepted threshold values, and concerns over cost-effectiveness hinder broad implementation. Furthermore, the multifactorial etiology of diabetes necessitates a systems biology approach, integrating cytokine profiles with genetic, metabolic, and clinical data.

In conclusion, interleukins serve as both mechanistic contributors and potential clinical tools in diabetes. Their incorporation into personalized care models may enhance the early detection of complications and enable targeted immunomodulation to improve long-term outcomes.

## 6. Conclusions

This review underscores the complex and multifaceted roles of interleukins in the immunopathogenesis of type 1 and type 2 diabetes, highlighting both their diagnostic and therapeutic potential. Interleukins such as IL-1β, IL-6, and IL-10 emerge as central mediators in β-cell dysfunction, chronic inflammation, insulin resistance, and the progression of diabetes-related complications. However, their pleiotropic nature presents a significant therapeutic challenge. For instance, while IL-1β inhibition has demonstrated beneficial effects in improving glycemic control and preserving β-cell function, it also carries risks related to immunosuppression and increased susceptibility to infections. Similarly, targeting IL-6 may interfere with essential physiological processes such as hematopoiesis and acute-phase responses.

A critical translational barrier is the dual—or even opposing—functions of certain cytokines depending on the disease stage, cellular context, and tissue microenvironment. These complexities necessitate a more refined understanding of cytokine isoforms, receptor subunits, and intracellular signaling cascades. For example, the differential roles of IL-1 receptor antagonist (IL-1Ra) in modulating immune responses remain poorly characterized and warrant deeper investigation, particularly in human models. Furthermore, the interplay between pro- and anti-inflammatory interleukins in shaping tissue-specific immune tolerance, especially in pancreatic islets, adipose tissue, and the kidney remains inadequately defined.

Future research should address the following gaps: (1) longitudinal human studies validating interleukin signatures as predictive biomarkers; (2) isoform-specific functional studies, particularly for IL-1Ra, IL-6R, and IL-10; (3) trials evaluating combinatorial cytokine targeting to reduce redundancy and compensation; and (4) the development of tissue- or cell-specific delivery systems to minimize off-target effects.

In conclusion, while interleukins offer a promising avenue for early diagnosis and immunomodulatory therapy in diabetes, their successful clinical application will depend on resolving key mechanistic ambiguities, optimizing safety profiles, and ensuring translational relevance across diverse patient populations.

## Figures and Tables

**Table 1:** Main findings of interleukins on type 1 and type 2 diabetes.

Interleukins	Main Findings from Clinical Studies	
	T1D	T2D
Interleukin-1	Elevated IL-1β levels suggest its utility as a biomarker of disease activity and progression [14,15,16]. IL-1β contributes to the destruction of pancreatic β-cells by inducing nitric oxide-mediated necrosis, leading to autoimmunity [17].	IL-1β is primarily induced by chronic hyperglycemia resulting in β-cell apoptosis and reduced insulin secretion [18].
Interleukin-2	IL-2 levels were high in T1D, which may reflect an early immune imbalance, potentially before the onset of T1D, supporting its role as a marker of immune dysregulation [19].	Suri et al. [20] observed anti-inflammatory IL-2 activity in newly diagnosed T2D patients, pointing to its potential as a diagnostic biomarker.
Interleukin-3	T1D patients have weakened hematopoietic responses to IL-3 and GM-CSF, suggesting a shared dysfunction in these pathways that may contribute to disease development [21].	Unknown role yet.
Interleukin-4	Reduced IL-4 in T1D patients at disease onset could contribute to unrestricted inflammation within the pancreatic islets, thereby accelerating the autoimmune process [22].	A positive correlation between IL-4 in T2D diabetic patients contributes to metabolic inflammation and insulin resistance [23].
Interleukin-5	Unknown role yet.	Elevated IL-5 levels could even serve as a biomarker for brain-related complications in diabetes and also contribute to neuroinflammation [24].
Interleukin-6	Elevated IL-6 levels have been observed in T1D patients that may contribute to autoimmunity [25].	Elevated IL-6 levels, contributes to insulin resistance, by interfering with insulin signaling and promoting inflammatory responses [26].
Interleukin-7	Elevated IL-7 levels suggest a possible delayed effect or compensation mechanism in chronic disease [27].	Unknown role yet.
Interleukin-8	Elevated IL-8 levels could be used as a predictive marker of disease progression or severity in T1D [28].	Elevated IL-8 levels suggested as a biomarker of poor metabolic control and also increased risk of diabetic complications [29].
Interleukin-9	Elevated IL-9 levels correlated with the albumin-to-creatinine ratio, suggesting it as a marker to detect early signs of kidney stress in T1D [30].	Lower IL-9 levels suggest a regulatory or protective role in the later stages of kidney disease [31].
Interleukin-10	Elevated IL-10 levels lead to severe presentations such as diabetic ketoacidosis, suggesting that high IL-10 could reflect more active or prolonged inflammation in younger individuals [32].	IL-10 gene has been linked to higher T2D risk, possibly through its impact on insulin signaling and immune regulation [33].
Interleukin-12	1159A allele is linked to increased gene expression, suggesting that elevated IL-12p40 levels may contribute to the genetic risk for T1D [34].	High IL-12 levels, strongly linked to insulin resistance, were also associated with chronic inflammation and various health conditions [35].
Interleukin-17	Elevated levels of Th17 cells and IL-17 in children with newly diagnosed T1D and IL-17 enhanced inflammatory signaling and increased pro-apoptotic responses in human islet cells, suggesting a direct contribution of IL-17 to islet cell destruction in the early stages of the disease [36].	Higher IL-17 levels in T2D patients, are connected to both inflammation and metabolic problems [37].
Interleukin-18	Higher IL-18 levels involved active inflammation then long-term tissue damage [38].	Higher IL-18 levels may actively contribute to the disease’s pathogenesis [39].
Interleukin-23	Elevated IL-23 suggests imbalance linked with poor lipid profiles [40].	Elevated IL-23 levels suggest a role in low-grade systemic inflammation [41,42].
Interleukin-27	myeloid dendritic cells showed increased IL-27 receptor expression and stronger downstream signaling, along with high levels of inhibitory checkpoint molecules like PD-L1 and PD-1 [43].	Early T2D may involve immune dysregulation due to insufficient IL-27, while later stages involve compensatory inflammation [44].
Interleukin-33	IL-33 increases Tregs and FOXP3 expression in T1D patients, suggesting IL-33’s potential in enhancing immune tolerance and serving as a target for immunotherapy [45].	T2D patients have lower serum IL-33 and higher soluble ST2 (sST2) levels compared to healthy controls, with IL-33 negatively correlating with blood glucose levels. This suggests that IL-33 might be linked to better glucose control and insulin sensitivity, independent of lipid levels [46].
Interleukin-35	Low IL-35 levels with more severe T1D and greater β-cell destruction; and significantly lower IL-35 levels and reduced populations of IL-35+ immune cells (Tregs, Bregs, and CD8+FoxP3+ cells) [47].	IL-35 can reduce diabetic neuropathic pain (DNP) by lowering inflammation and nerve cell death in the spinal cord, likely through the inhibition of the JNK pathway, a major signaling route involved in inflammation and pain perception [48].

## Data Availability

No datasets were generated or analyzed during the current study. All tables and figures in this manuscript were created by the authors and are not subject to any copyright restrictions.
